# Experiences of professional support during pregnancy and childbirth – a qualitative study of women with type 1 diabetes

**DOI:** 10.1186/1471-2393-9-27

**Published:** 2009-07-03

**Authors:** Marie Berg, Carina Sparud-Lundin

**Affiliations:** 1 The Institute of Health and Care Sciences, the Sahlgrenska Academy at Gothenburg University Box 457, SE-405 30, Göteborg, Sweden

## Abstract

**Background:**

Women with type 1 diabetes are at high risk of complications during both pregnancy and childbirth. Stringent monitoring of blood sugar is required in order to improve the chance of giving birth to a healthy child; however, this increases the incidence of severe hypoglycaemia. The aim of this study was to explore the need for and experience of professional support during pregnancy and childbirth among women with type 1 diabetes.

**Methods:**

The study has a lifeworld research approach. Six focus groups and four individual interviews were conducted with 23 women, 6–24 months after delivery. The participants were encouraged to narrate their experiences of pregnancy and childbirth in relation to glycaemic control, well-being and provided care. Data analysis was directed towards discovering qualitative meanings by identifying and clustering meaning units in the text. Further analysis identified eight themes of meaning, classified under pregnancy or childbirth, forming a basis for a final whole interpretation of the explored phenomenon.

**Results:**

The women felt worry about jeopardizing the baby's health and this was sometimes made worse by care providers' manner and lack of competence and support. The increased attention from care providers during pregnancy was experienced as related to the health of the unborn child; not the mothers. Women who during pregnancy received care in a disconnected diabetes organisation were forced to act as messengers between different care providers.

**Conclusion:**

Clarity in terms of defining responsibilities is necessary during pregnancy and childbirth, both among care providers and between the woman and the care provider. Furthermore, a decision must be made concerning how to delegate, transfer or share diabetes responsibility during labour between the care providers and the parents-to-be.

## Background

Pregnant women with diabetes are at high risk of complications [1,2] and at increased risk of adverse childbirth outcomes, such as fetal congenital abnormalities, macrosomia, obstetrical and neonatal complications. There is a strong relationship between good glycaemic control and better outcomes [3-6], and stringent glycaemic control beginning with planning pregnancy and throughout pregnancy and labour, is thus required. However, this increases the incidence of severe hypoglycaemia [7,8] and there is still a knowledge gap regarding how the benefits of strict glycaemic control balance the risks of severe hypoglycaemia [9].

Pregnant women at high risk, as in the case of diabetes, feel more anxiety, worry and ambivalence than those with low-risk pregnancies [10]. A study of experiences of pregnancy [11] showed that daily lives for women with type 1 diabetes were characterized by exaggerated feelings of responsibility and perceived demands from the child, as well as constant worry, self-blame and pressure to provide the child with the best conditions to enable being born healthy; they felt 'ruled' by their blood glucose. The body was perceived as changed and symptoms of objectification, including loss of control, appeared. Feelings of unfamiliar body responses and unpredictability due to an increased number of hypoglycaemic episodes and the inability to recognise one's hypoglycaemic episodes were described in a study performed in Australia [12]. Moreover, the need to achieve good glycaemic control can construct a duality 'to be master or be enslaved' as the pregnant woman deals with life. The care providers' manner either supports the feeling of mastership or of slavery [13]. Research on how women with diabetes perceive support received during pregnancy and childbirth is limited. Thus, the aim of this study was to explore the need for and experience of professional support during pregnancy and childbirth among women with type 1 diabetes.

## Methods

### Design

The study was approved by the Regional Ethics Board (Dnr: 351-07). It was conducted with a reflective, hermeneutic, lifeworld research approach, emphasising that understanding of human beings' lifeworlds is necessary to grasp how they relate to and interact with the world. A person's lifeworld is constituted by the past, present and future; all experience is embodied and the surrounding world is inseparable from the body [14]. The challenge in the lifeworld research approach is to have an open stance including bridling one's own pre-understandings, sensitive and pliable to the studied phenomenon with the aim of analysing its meaning [15].

### Setting, participants and data collection

The prevalence of type 1 diabetes in mothers of newborns in Sweden is about 0.3–0.4%. In this study, mothers with type 1 diabetes living in the western region of Sweden were invited to participate in focus groups (FG). The region has four hospitals with maternity units, managing an annual total of 16000 deliveries at the time of the study with women living in both rural and urban areas. The overall objectives for the care of pregnant women with type 1 diabetes in the region are prevention, early detection and treatment of maternal and fetal complications as well as the provision of professional support to enhance safety, continuous education and encouragement to the pregnant women and her relatives. The current regional diabetes pregnancy care program includes detailed guidelines related to diabetes severity and pregnancy complications. However, antenatal care models in the region differed (see Table 1), although the secondary antenatal clinics all had a multidisciplinary approach. 

**Table 1:** Study group characteristics (n = 23 mothers)

Mother's age at delivery	32 (22–37)*
Educational level	
Secondary level	11
University	12
Occupation	
Health care	9
Trading	4
Administration/Communication	5
Education	3
Cleaning	1
Student	1
Parity	
Primiparous	13
Multiparous	10
Child's age at interview (months)	11 (6–24)*
Antenatal care level	
Exclusively secondary antenatal clinic	17
Combined primary and secondary antenatal clinics**	4
Combined primary and secondary antenatal clinic,	
primary diabetes care**	2
Delivery mode	
Spontaneous vaginal	10
Vaccum extraction	0
Elective caesarean section	1
Acute caesarean section	12

* Median (range)

** Primary antenatal clinic = general maternal health care clinic

Secondary antenatal clinic = Antenatal clinic for women with high obstetric risks

Primi- and multiparous Swedish-speaking mothers with an interval of at least 6 months since delivery were recruited from the four hospitals' delivery wards. The purpose was to obtain reports of varied experiences of received care and support. A list of mothers was given to the first author who contacted them with written and verbal information about the study. FGs can generate rich data through the interaction created between the participants, enabling a clarification of both similarities and differences in experiences through a sharing, acquiring and contrasting process [16,17].

Twenty-five mothers agreed to participate in a FG, and six failed to take part for last-minute reasons. Of these six, two women were interviewed individually as they could not participate due to own ill health (one) or the child's ill health (one). Two other women preferred to be individually interviewed due to distance (one) and a desire for more privacy (one). Finally, six FGs, including 19 women and four individual interviews were performed with a total of 23 women who gave written informed consent to be interviewed. The FGs included two to five participants, lasted 90 to 120 minutes and were performed at three of the hospitals. The individual interviews lasted 25 to 60 minutes; three were performed by telephone and one face-to face at the fourth hospital. All interviews were audio-recorded and transcribed verbatim.

Both researchers (CSL and MB) were present during the interviews, one acting as moderator and the other taking field notes on the interactions and asking complementary questions [16]. The participants were asked to introduce themselves and encouraged to narrate their experiences of pregnancy and childbirth in relation to glycaemic control, well-being and provided care. During the discussions, the women inspired each other to recall and reflect on both similar and contrasting situations and feelings related to their experiences. They were encouraged to describe their own experiences and to feel free to agree or to present experiences that contrasted with those recounted by the other participants. The moderator posed open and clarifying questions in accordance with the lifeworld perspective, e.g. 'Can you tell us about your experience during labour?', 'Can you tell us about your experience of the support you received, or the lack of support?' and clarifying questions, such as: 'Can you elaborate on that?', 'What did you feel?', in order to enrich the stories. The individual interviews were conducted with the same open questioning.

### Data analysis

The interpretative analysis of all transcribed text was directed towards discovering varied qualitative meanings of the phenomenon 'the need for and experience of professional support in relation to glycaemic control during pregnancy and childbirth among women with type 1 diabetes'. No predetermined hypotheses, theories or interpretive sources were used. An intensive dialogue with the text was undertaken, in which the whole was understood in terms of details and details in terms of the whole. Meaning units were identified in the text and clustered. In the further analysis, themes of meaning were recognized, forming a basis for a final whole interpretation of the explored phenomenon [15].

## Results

Characteristics of the participating women, antenatal care levels and modes of delivery are presented in Table 1. The duration of the type 1 diabetes varied between 4 and 31 years (mean 15 years); most women administered insulin by pen, and some by pump. The need for and experience of professional support in relation to glycaemic control is described in eight themes of meaning which are presented below, classified under one of the two domains pregnancy or childbirth, and exemplified in quotes marked as individual interview (I:1–4) or FG (FG:1–6). Individual women are not identified. An overview of the themes of meaning and final interpretation is presented in Table 2.

**Table 2:** Overview of themes of meaning and final interpretation

**A need to clarify responsibility**	
• Feeling pressure*	• Feeling abandoned, left with responsibility for glycaemic control**
• Carrying a child gives you priority*	• Needing to stay in control**
• Advice occasionally unreliable*	• Both trust and distrust**
• Being a messenger in a disconnected care organisation*	
• Needing to share experiences*	

* Themes of meaning during pregnancy

** Themes of meaning during childbirth

### Pregnancy

The women had to different degrees, before and during pregnancy, been prepared by the care providers for the need to achieve normoglycaemia. The participants had experienced this preparation differently. One mother described how she had been told by her physician at age 18 about the importance of satisfactory glycaemic control when planning pregnancy, and how this had influenced her preparation for and self-management during pregnancy: '*I was very careful about that when we were planning to get pregnant. And then I felt great during the entire pregnancy and was really careful about my glucose levels, which I'm not always otherwise' *(FG3).

#### Feeling pressure

The pregnancy generated different degrees of pressure. The driving force was the baby's well-being which required frequent blood glucose tests and calculation of insulin boluses in order to achieve optimal glycaemic levels. The majority had had to increase insulin dosages successively as pregnancy progressed, but some women had instead had to lower their total amount of insulin. The struggle for optimal blood glucose levels had led to serious hypoglycaemic symptoms for some women; one had driven off the road twice, another had suffered unconsciousness with seizures and had required help from the ambulance team to attain sufficient oxygen levels, while a third woman had felt a strong fear of dying:

*And sometimes in the evenings I was like...I don't know if I dare go to bed, what if I die in my sleep and my husband doesn't notice anything? That really gives you a lot of anxiety, you don't know how you're going to survive*. (FG4)

Pregnancy was characterised by constant worry for the baby, influenced by the hypoglycaemic episodes and undulating blood glucose levels and thoughts about the consequences for the baby. A prevailing worry about increased fetal growth and the risk of more difficult labour with maternal and neonatal complications was experienced. Information given by care providers could contribute to and increase worry. For example, one midwife had said that '*a large baby is not a strong baby, it's a large and fragile baby' *(FG3). One woman had had exaggerated conceptions of how dangerous hyperglycaemia during labour could be for the baby. Another woman had feared that the baby could become comatose and even risk dying if she were hypoglycaemic after birth. Emphasis on the increased risks had created feelings of guilt in some women.

*I think it wasn't until my third or fourth month that my HbA1C levels started going down to 5,1–5,2*. *And the way they kept talking about that there was a very, very, very major focus on my having to lower my levels because it's very dangerous for the baby*. (I1)

#### Carrying a child gives you priority

The women felt prioritized by the care providers during pregnancy compared to ordinary diabetes care, both in terms of access, competence and attention. Professional support entailed an established, trustful relationship. One woman who had attended both an ordinary primary antenatal clinic and a secondary, specialised antenatal clinic appreciated being treated both as an ordinary pregnant woman and as a mother-to-be in need of specific diabetes-related competence. A generous attitude toward sick leave during pregnancy was highly appreciated, as this was often compulsory in order to achieve strict glycaemic control. The increased attention from care providers was experienced as related to the baby in the womb; his/her health, not the mother's, was given the highest priority.

*And then that's how it felt, is it just because I'm carrying a baby, because otherwise they don't care, or they don't care about me. (FG2)//And that's how it is, isn't it? And it's very, like, here you are, you're pregnant and we're focusing on the baby*. (I1)

This was especially obvious in the case of one participant who had given birth to two children before the diabetes onset and could thus compare experiences during those two pregnancies with her third pregnancy. Another woman had had severe problems maintaining optimal glycaemic control. Many hypoglycaemic episodes created feelings of irritability and depression, and she perceived that the care providers did not take these negative effects on her well-being into consideration. The feeling of not being in the focus of the care was verified after the birth of one mother's baby as the care immediately became less intensive:

*It's such an enormous difference. Because the mum is used to being really well looked after...and then 'poof'' it's gone*. (I4)

#### Advice occasionally unreliable

The women had noted a generally high level of competence concerning diabetes among the care providers but some had also experienced insufficient professional competence, including either incorrect management or no management at all. This seemed to increase the feeling of pressure and could for example concern not receiving answers to questions, leading to a need to act as one's own expert. One woman had drawn the conclusion that there were no guidelines on treatment of pregnant women with diabetes; another woman had had this feeling in connection with hospital treatment of a diabetes-related complication. A third woman had experienced the following at a visit to the primary antenatal clinic:

*My midwife would make the same recommendation several times, for instance that I should take my iron tablets with a glass of juice. And I told her I couldn't do that. So we went out and took my blood count and it was a bit low and then she repeated it, 'Take your iron with some juice.', and I said, 'I still can't do that because my blood glucose will go over the top.', and then she said, 'Oh, yes, right!'*. (FG5)

#### Being a messenger in a disconnected care organisation

Care organisation during pregnancy differed; i.e. no universal routine existed. In two of the hospitals, diabetic women were exclusively cared for at the hospitals' specialised, secondary antenatal clinics for high-risk mothers, including regular and frequent visits to a midwife specialised in diabetes and to an obstetrician who consulted a diabetologist as needed. Women belonging to the two other hospitals' catchment areas had received a disconnected type of care, the most extreme of which entailed ordinary follow-ups with a midwife at the primary antenatal clinic, blood glucose checkups at the diabetes clinic and diabetes- related visits with both midwife and obstetrician at the specialised, secondary antenatal clinic. Furthermore, all women saw other professionals, such as dieticians and ophthalmologists. Experience of disconnected care is expressed in the following:

*I went in to my ordinary antenatal clinic in my hometown, for ordinary antenatal care, because you didn't get much of that here *(at the hospital). *So I had the midwife there for another type of support, advice and other ordinary things. I came here more to see doctors and get checked up with scans and things like that. So it was very divided*. (FG5)//*So you went in to see your ordinary midwife and the next day you had to go in to the specialist clinic at the hospital and then the day after that perhaps you had to see the internist. There were so many trips back and forth, an awful lot of travelling about*. (FG6)

The number of visits, including to different specialists, increased as pregnancy proceeded. This was particularly difficult for women who were not on sick leave or who lived far from the different care providers. Another problem was insufficient communication between the different care providers; it was often unclear who was responsible for what. This sometimes provided the pregnant women with more questions than answers and burdened them with the role of messenger, reporting which follow-ups and treatments had been performed or planned in the different care settings:

*It was like you had to run around with a lot of papers. And like I, with no training in any health care profession, was expected to know which tests were to be taken at which week. That isn't easy*. (FG5)//*Then they asked me, 'Have you thought about pain relief?'. So I brought that up when I was talking to her and she said that I had to talk about that issue elsewhere and when I brought up another question they said, 'You have to take that up with them instead.' *(FG5)

#### Needing to share experiences

The women had a need to share experiences with other pregnant women with diabetes; some expressed feelings of loneliness as pregnant diabetics. This became obvious after finishing the FG discussions; the participating women continued to talk with each other and exchanged phone numbers. A few had taken the initiative to seek support on websites. It was proposed that care providers could support this type of communication, for example, by arranging group meetings for pregnant diabetics or by distributing addresses:

*When I talk to my friends who also have children, they can complain a lot about having had troublesome pregnancies, and so on. Of course, that's how they feel, isn't it, but you're quite alone, aren't you....as a pregnant diabetic? *(FG2)

### Childbirth

#### Feeling abandoned, left with responsibility for glycaemic control

During labour, a feeling of being abandoned by the midwife, when it came to glycaemic control, was apparent. This was connected with lack of or insufficient information concerning care routines, often with the implicit expectation that the woman herself should take the frequent glucose tests required during labour, and sometimes even adjusts the insulin doses if needed. The father-to-be often became responsible for blood glucose tests, reducing his possibility to follow the flow of labour.

*My husband has never checked my blood sugar. Perhaps it was stupid not to tell him how to do it, but I'm a bit of a control person so I do it myself. And it works just fine. But then you're in this situation and he's very stressed because he's meant to be take charge. I really felt sorry for him, but at the same time, I'm lying there with contractions and I have to try to be nice to him and show him, and so on... Actually, I thought that they'd help us with that*. (FG3)

One woman and her partner had especially asked to be relieved of the responsibility of blood glucose control during labour. Nevertheless, they had been forced to undertake it and their disappointment was still very central. '*and we're very dissatisfied because we had written in all our papers that we didn't want to do that. We wanted to concentrate on having the baby.' *(FG 2). This feeling of having the utmost responsibility for glucose level control during labour was associated with severe worry in several women. One woman felt strong anxiety and uncertainty when her blood glucose levels rose, feeling that the care provider took no notice of the high levels and thus could not reassure her. Another woman felt abandoned when it came to management of the insulin pump. Still dazed after a caesarean section, she had to change her insulin pump catheter as the care providers had blocked the catheter with tape when preparing for surgery and no one knew how to handle it.

#### Needing to stay in control

On the other hand, some women felt a need to monitor blood glucose levels and control insulin adjustments during childbirth as it gave them a sense of security: *'I'm a bit of a control freak so I'd rather do it myself and know what's going on. To check my level and know what it means instead of having to explain to a lot of people and all that kerfuffle' *(FG5). Control could also be maintained by letting the partner check blood glucose levels. This could increase his participation and collaboration with the midwife, and the woman could focus on giving birth. Worry about losing control of one's diabetes was also reported, illustrated in the following quote from a woman who wanted to stay in control, although she did not understand how this would be possible during labour: '*I had asked them during labour, how the dickens am I going to cope with the whole blood sugar business?' *(FG3). Her narrative also indicates that she had not obtained correct information regarding the impact of blood glucose levels on the baby's health: *'I couldn't accept losing control of my blood sugar so that she'd start life with her sugar too low, in a coma. So I felt it was really, really dangerous when that happened.' *(FG3)

#### Both trust and distrust

Both trust and distrust in care providers' specific diabetes-obstetric competence were described in the interviews. On the one hand, a kind of 'institutional trust' was expressed, illustrated in the following focus group discussion:

Woman 1: *I wasn't worried about having high levels. Maybe I didn't know enough about the baby ending up in a coma like you said*.

Woman 2: *I've never heard anything about that happening during delivery, perhaps I never asked either. I mean, when you're in hospital, like you said, you're not in Africa, they take care of everything*.

Woman 3: *Yes, that's what I was thinking. Your blood sugar might be low but then I guess they'll put my baby on a drip or help me out somehow*. (FG3)

On the other hand, distrust in care providers' knowledge of how to handle blood glucose levels created feelings of insecurity and escalating stress during as labour progressed. This could result from being given incorrect advice or instructions or from absent reactions to high blood glucose levels as a consequence of a glucose infusion administered early. One woman who was a nurse, knowing how labour should be managed, had been given totally wrong orders:

*So I knew a bit about insulin drips: rapid-acting insulin is added to the glucose drip and it should preferably be given by pump and so on. And then this house officer tells me to take half my long-acting insulin dose and add it to the glucose drip. So there you are, with your sick bag and having contractions and all that. 'You mustn't do that, you mustn't do that, call my internist! Don't give me long-acting or we'll lose control of my sugar levels.' And so finally I convinced them to give me a glucose drip and my husband had to check my sugar and I gave myself insulin by pen as I went along. And you're not in such great shape yourself at that point*. (FG4)

### Final interpretation: a need to clarify responsibility

For these women with type 1 diabetes, the overall goal of giving birth to a healthy baby required frequent blood glucose checks and insulin adjustment. Worry about jeopardizing the baby's health was central and was sometimes made worse by care providers' manner and lack of support. Professional competence in managing diabetes in pregnancy and childbirth was essential to these women. In general, they appreciated this competence although they felt that attention was primarily focused on the baby's health, sometimes at the expense of their own wellbeing. Experiences of distrust and of being abandoned were reported. When care during pregnancy was divided according to care providers' specialities, the women was treated as a disjointed entity and burdened with the role of messenger between the different care providers. During labour, a common professional approach was to let the women be responsible for glycaemic control, i.e. blood sugar testing and insulin adjustment. Some preferred this as it gave them a sense of control, while others felt abandoned and wanted to concentrate on the birthing process itself by being relieved of responsibility. A trustful relationship, in which the woman feels prioritized for her own sake and well-being as well, appears to be crucial in supportive professional care. In summary, it seems to be a question of clarifying responsibility, both among care providers as well as between the woman and the care provider, for instance during childbirth, during which the basic goal should be to support the woman in following the flow of labour and birth, only being responsible for glycaemic control on her own terms.

## Discussion

The final interpretation of the results of this study revealed a need to clarify responsibility for diabetes during pregnancy and childbirth, both between different care providers and between the care provider and the woman/her partner. Although diabetes care providers have the medical responsibility to minimize adverse outcomes of pregnancy, women with type 1 diabetes must, and want to, cope with the need for strict glycaemic control. In earlier research, this has been described as '*being in the grip of blood glucose levels*' [18] (page 300), and as '*being controlled by the blood glucose levels for the child's sake*' [11] (page 39).

A noteworthy negative experience during pregnancy reported in our study was to be a messenger between different care providers, prominent in disconnected care organisations, which also led to experiences of distrust and uncertainty concerning the professionals' diabetes competence and a perceived need for the woman to serve as her own expert. This echoes other research showing that women were less satisfied with the support provided by care providers with limited experience of and knowledge gaps concerning diabetic pregnancies [12]. A care organisation in which the woman is required to act as a messenger does not provide 'good quality of care'. To be in transition to motherhood requires a supportive environment, particularly when a mother-to-be is at high risk. In general, the women in our study felt prioritized as receiving more diabetes support during pregnancy than ever else, which can be considered as "high marks" for diabetes midwives and physicians. Local differences in access to specialist antenatal care, as found in this study in which the women had been given care in four different settings, seem to explain the negative consequences of the disconnected model of care. Based on our findings, we suggest that a multi-professional team should manage care of pregnant women with diabetes, in rural as well as urban areas, with as few care providers as possible. Moreover, in line with Lavender et al. [19] we suggest the existing diabetes pregnancy care programs to be more extensive concerning psycho-social support. All women, both from urban and rural areas, in our study as well as in others [12,18] wanted contact with others with similar experiences during pregnancy and childbirth. Websites offering both professional support and support from women in similar situations (via chats and forums) might alleviate the effects of insufficient access to face-to-face professional care.

Our findings concerning the women's experience of childbirth complement earlier studies. In order to decrease feelings of vulnerability, uncertainty and guilt in women with diabetes, we suggest that care during childbirth include support for the women to stay in control [18]. Based on our findings, we suggest that this should include relieving them of 'being in charge' when they need and wish for such relief. The respective, and contrasting, desires to stay in control and to be relieved of control during labour and childbirth place high demands on care providers to be sensitive and flexible towards the woman's and her partner's needs. The feeling of abandonment during childbirth expressed by some women might have been avoided by mutual agreement and the clarification of responsibility, for instance by a written birth plan. At admission to the labour ward the midwife might, for example, ask, 'How would you like things done? Do you want to check your blood glucose or just leave it to us?'

It is well known that negative birth experiences are associated with women's worries regarding their own and the baby's health during pregnancy [20], and that worry is more common among women at high risk, including women with diabetes [10,21]. Therefore, attentiveness in midwives and obstetricians to the individual needs of the woman and her partner is crucial. This should include participation in decision-making and supportive behaviour as it has been shown to reduce negative experiences [20].

### Methodological considerations

Combining FG discussions and individual interviews was not a decision made beforehand. Instead, the aim was to explore the phenomenon in the best possible way, while respecting individual preferences. Of course it can be questioned if it is possible to acquaint oneself with individual lifeworlds in a FG in which several lifeworlds are encountered and mixed, sometimes creating collective meanings.

Concerning the trustworthiness of our study we aimed at using the interaction effects related to the FGs by means of an additional layer of data. This is enhanced by the social space that FGs provide, in which the participants view and experience constructs through evolving discussions and interactions [17]. However, in order to reach the full potential of FGs it is essential to pay particular attention to analysis of the unique effects of the interaction [22]. In this study, such insights were obtained in some FGs more than in others, probably related to the atmosphere and personalities combined in each constellation. Similar to the descriptions by Lambert & Loiselle [23], different patterns were illuminated and discussed in each group according to what was experienced and relevant in that FG context. During the individual interviews, on the other hand, the woman's experience was expressed without being contrasted and reflected upon by others, occasionally leading to more detailed and rich descriptions. Thus, the methods complemented each other, yielding differing layers of data. In the analysis, both individual and contextual interpretations of the phenomenon were developed in the integration of data. It is essential to critically scrutinize strengths and weaknesses pertaining to both data collection methods, while acknowledging that the combination of methods can create a better understanding of different representations of the phenomenon [23].

Another credibility issue is the authors' proximity to the research field; one of us (MB) has extensive experience as a "diabetes midwife" and the other (CS-L) as a neonatal nurse, both at one of the four included hospitals. However, we did not treat any of the included participants during the study period. We are aware that closeness to a study phenomenon may have an impact on the data but we consider this to be an asset, rather than a problem, especially when applying a hermeneutical approach, which is based on the assumption that there can be no understanding without pre-understanding. In order to achieve scientific reliability, we have critically examined all developed interpretations, including our own pre-understandings related to the studied phenomenon, until we reached the final interpretation [15].

Regarding the transferability of our findings, the participants represent a panorama of women with type 1 diabetes in the region; they come from both rural and urban areas and have been provided with a variation of antenatal care models, depending on which health care institution they belong to. Their educational level is fairly comparable to that in a normal population.

## Conclusion

All human beings are unique, including women with type 1 diabetes; it is therefore difficult to create a model of care that suits everyone in this group. Establishing a trustful relationship in which the woman feels prioritized also for her own sake and well-being, not only for the baby's sake, appears to be crucial for supportive professional care. No matter how the individual women's preferences might vary, deciding in advance how to delegate, transfer or share responsibility for managing the diabetes during labour seems to be a challenge in clinical practice. There is an obvious need for clarity concerning who is responsible for what, both among care providers and between the woman and the care provider, during pregnancy and childbirth. We might have to convince the woman and her partner that the professionals are there for them, ready to relieve them of the burden of staying in control if that what is needed. This also includes introducing the women to others in the same situation as they can support each other and confirm one another's experiences, complementing the support provided by professionals.

## Competing interests

The authors declare that they have no competing interests.

## Authors' contributions

Both authors contributed to all stages of the research, revised the manuscript and wrote the final version of the paper.

## About the authors

The authors work at the Institute of Health and Care Sciences, the Sahlgrenska Academy at Gothenburg University Box 457, SE-405 30, Gothenburg, Sweden. MB is Associate Professor, RN and RM, and CSL is PhD and RN.

## Pre-publication history

The pre-publication history for this paper can be accessed here:



## References

[B1] Diabetes care and research in Europe (1990). the Saint Vincent declaration. Diabet Med.

[B2] Kinsley B (2007). Achieving better outcomes in pregnancies complicated by type 1 and type 2 diabetes mellitus. Clin Ther.

[B3] Platt MJ, Stanisstreet M, Casson IF, Howard CV, Walkinshaw S, Pennycook S (2002). St Vincent's Declaration 10 years on: outcomes of diabetic pregnancies. Diabet Med.

[B4] Evers IM, de Valk HW, Visser GH (2004). Risk of complications of pregnancy in women with type 1 diabetes: nationwide prospective study in the Netherlands. BMJ.

[B5] Nielsen GL, Moller M, Sorensen HT (2006). HbA1c in early diabetic pregnancy and pregnancy outcomes: a Danish population-based cohort study of 573 pregnancies in women with type 1 diabetes. Diabetes Care.

[B6] Temple RC, Aldridge VJ, Murphy HR (2006). Prepregnancy care and pregnancy outcomes in women with type 1 diabetes. Diabetes Care.

[B7] ter Braak EW, Evers IM, Willem Erkelens D, Visser GH (2002). Maternal hypoglycemia during pregnancy in type 1 diabetes: maternal and fetal consequences. Diabetes Metab Res Rev.

[B8] Rosenn BM, Miodovnik M (2000). Glycemic control in the diabetic pregnancy: is tighter always better?. J Matern Fetal Med.

[B9] Evers IM, ter Braak EW, de Valk HW, Schoot B van Der, Janssen N, Visser GH (2002). Risk indicators predictive for severe hypoglycemia during the first trimester of type 1 diabetic pregnancy. Diabetes Care.

[B10] Gupton A, Heaman M, Cheung LW (2001). Complicated and uncomplicated pregnancies: women's perception of risk. J Obstet Gynecol Neonatal Nurs.

[B11] Berg M, Honkasalo ML (2000). Pregnancy and diabetes–a hermeneutic phenomenological study of women's experiences. J Psychosom Obstet Gynaecol.

[B12] King R, Wellard S (2009). Juggling type 1 diabetes and pregnancy in rural Australia. Midwifery.

[B13] Berg M (2005). Pregnancy and Diabetes: How Women Handle the Challenges. J Perinat Educ.

[B14] Merleau-Ponty M (1974). Phenomenology of perception.

[B15] Dahlberg K, Dahlberg H, Nyström M (2008). Reflective lifeworld research.

[B16] Krueger RA, Casey MA (2009). Focus groups: a practical guide for applied research.

[B17] Lehoux P, Poland B, Daudelin G (2006). Focus group research and "the patient's view". Soc Sci Med.

[B18] Rasmussen B, O'Connell B, Dunning P, Cox H (2007). Young women with type 1 diabetes' management of turning points and transitions. Qual Health Res.

[B19] Lavender T, Platt MJ, Tsekiri E, Casson I, Byrom S, Baker L (2009). Women's perceptions of being pregnant and having pregestational diabetes. Midwifery.

[B20] Waldenstrom U, Hildingsson I, Rubertsson C, Radestad I (2004). A negative birth experience: prevalence and risk factors in a national sample. Birth.

[B21] Berg M, Lundgren I, Lindmark G (2003). Childbirth experience in women at high risk: is it improved by use of a birth plan?. J Perinat Educ.

[B22] Freeman T (2006). 'Best practice' in focus group research: making sense of different views. J Adv Nurs.

[B23] Lambert SD, Loiselle CG (2008). Combining individual interviews and focus groups to enhance data richness. J Adv Nurs.

